# One pot fabrication of fluorescein functionalized manganese dioxide for fluorescence “Turn OFF–ON” sensing of hydrogen peroxide in water and cosmetic samples†

**DOI:** 10.1039/d0ra01980a

**Published:** 2020-05-05

**Authors:** Hassan Refat H. Ali, Ahmed I. Hassan, Yasser F. Hassan, Mohamed M. El-Wekil

**Affiliations:** Department of Pharmaceutical Analytical Chemistry, Faculty of Pharmacy, Assiut University Assiut 71526 Egypt mohamed.mohamoud@ymail.com; Department of Pharmaceutical Analytical Chemistry, Faculty of Pharmacy, Al Azhar University Assiut 71526 Egypt

## Abstract

In recent decades, H_2_O_2_ has been promoted as a health indicator because its moderate to high levels can cause some health problems. Herein, we developed a new fluorescent nanoprobe for rapid, selective and sensitive detection of H_2_O_2_. The fluorescent nanoprobe is composed of fluorescein dye (FLS) as a fluorescent probe and MnO_2_ nanosheets (MnO_2_ NS) as a quencher. In this study, H_2_O_2_ can reduce MnO_2_ NS in the synthesized composite and release FLS, causing sufficient recovery of fluorescent signal related to the concentration of H_2_O_2_. The nanoprobe, with *λ*_ex_/*λ*_em_ at 495/515 nm, has a linear range of 0.04–30 μM, with a limit of detection (LOD) of 7.5 nM and a limit of quantitation (LOQ) of 21 nM. The mean relative standard deviation (RSD) was 2.6% and the applicability of the method was demonstrated by the determination of H_2_O_2_ in water and cosmetic samples.

## Abstract 2

The fluorometric nanoprobe was fabricated *via* doping of fluorescein dye in MnO_2_ nanosheets (FLS/MnO_2_ NS) *via* facile co-precipitation method. It was used for analysis of H_2_O_2_ in different matrices through liberation of FLS after reduction of MnO_2_ NS.

## Graphical abstract

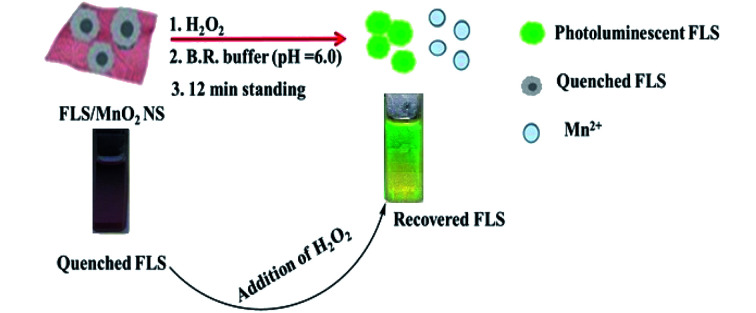

## 1. Introduction

Hydrogen peroxide (H_2_O_2_), a colorless liquid usually produced as aqueous solutions of various strengths, is used principally for bleaching cotton and other textiles and wood pulp, in the manufacture of other chemicals, as a rocket propellant, in cosmetics and for medicinal purposes.^1^

From a biological point of view, H_2_O_2_ is formed in humans and other animals as a short-lived product in biochemical processes and is toxic to cells. The toxicity is due to oxidation of proteins, membrane lipids and DNA by the peroxide ions, so it can be a serious health hazard as a high level of H_2_O_2_ can precipitate cancer in the duodenum of mice after drinking water administration at 0.1% (w/w).^2^

The systemic effects of H_2_O_2_ result from its interaction with the intracellular catalase enzyme accompanied by the liberation of oxygen and water upon its decomposition. One milliliter of 3% hydrogen peroxide liberates 10 mL of oxygen. When the liberated oxygen exceeds the maximum blood solubility, intravascular oxygen embolism may occur.^3^

Repeated exposures to hydrogen peroxide vapor may cause chronic irritation of the respiratory tract and partial or complete lung collapse. Also, inhalation or ingestion of high concentrations of hydrogen peroxide can result in seizures, cerebral infarction, or cerebral embolism that may end in permanent neurological deficits or death.^4^

Therefore, the analytical methodology must be available for the determination of H_2_O_2_ to investigate its physiological functions and diagnosing diseases.

Several methods have been proposed for estimation of hydrogen peroxide such as colorimetry,^5–7^ fluorimetry,^8–10^ chemiluminescence,^11,12^ chromatography^13–16^ and electrochemistry.^17–20^ Some of these techniques such as chromatography needs highly expert trainer, time consuming and require expensive instrumentation.

The integration of fluorescent nanoprobes and other effective nanostructures in a cross-linked matrix has been widely utilized in the fabrication of typical sensors. The quenching effect induced by nanomaterials towards up-conversion nanoparticles and luminescent probes has already been used to improve fluorescence sensing platform.^21–24^ Nevertheless, a crucial drawback of expanding the practical application of these sensing frameworks is the narrowed range of materials for switching the fluorescence of probes.

Manganese dioxide (MnO_2_) has possessed a considerable amount of current interest because manganese(ii) is the twelfth most common element on the planet and the third most abundant transition element after iron and titanium.^25^ Manganese(ii) ions act as cofactors in many functional enzymes with diverse mechanisms and a cornerstone in the oxygen-evolving units of photosynthetic tissues.^26^ Additionally, MnO_2_ has structural diversity; nanosheets, nanorods, nanospheres, nanobelts, nanowires, nanotubes, nanofibers and so on, which moreover expand its applications in a varied range of fields. Among the various MnO_2_ nanostructures, nanosheets provide adequate specific surface areas and high surface-to-volume ratios, permitting facile physicochemical interaction between reactants and its active sites. MnO_2_ NS can be internally reduced to Mn(ii), which in turn is considered to be friendly from the environmental and health points of view.^27^ It is worth to mention that, most of the reported MnO_2_ NS based fluorescent sensors offer some limitations such as an expensive reagent, time-consuming, low sensitivity, and poor MnO_2_ NS dispersity and complicated synthesized process.^28–32^

Fluorescein (FLS) FLS has attracted great interest in the fabrication of nanoprobes because FLS has been commercially available and so, avoiding complex preparation of emissive nanomaterials. Moreover, FLS has many functional groups that can be easily functionalized with MnO_2_ NS. Besides, the aqueous solubility of FLS enables the determination of several analytes in watery environment.^33^ FLS modified MnO_2_ NS was reported for analysis that relied on the distance between them, which was regulated with adsorption or desorption from MnO_2_ NS.^34^

In the proposed sensing system, we successfully synthesized MnO_2_ NS and fluorescein nanoprobe *via* a template-free, one step sonically treatment. The synthesized MnO_2_ NS, in turn quench the relative fluorescence intensity (RFI) of fluorescein dye through fluorescence resonance energy transfer mechanism (FRET).

In addition to being an efficient nanoquencher for the fluorescence nanoprobe, the synthesized MnO_2_ NS can also act as recognition agent for H_2_O_2_ as the latter can provoke the decomposition of the MnO_2_ NS which are selectively reduced into Mn^2+^, accompanying the dependent recovery of fluorescence intensity of FLS dye.

## 2. Experimental

### 2.1. Reagent and materials

Double distilled water (DDW) was used along the whole work. Fluorescein, glycine, ascorbic acid, sucrose, urea and ferric chloride were purchased from Alpha chemical, Mumbai. Cadmium nitrate and zinc sulfate were purchased from El-Nasr pharma, Egypt. Hydrogen peroxide, potassium permanganate and sodium thiosulfate were purchased from Adco pharma, Egypt. Maltose and copper sulfate were purchased from Lab Chemicals Trade – LCT, Egypt. Potassium hydrogen phosphate, magnesium chloride, calcium chloride, and ferrous sulfate were purchased from Oxford Laboratory Chemicals, India. Glutathione, cysteine, glucose and glucose oxidase enzyme R2 GOD 2701018 were purchased from Biomed Pharmaceutical industry, Egypt. Other reagents and chemicals were purchased from Modern Cairo For Chemicals - Chema Chems, Egypt. Oxygen water bottles (10%, v/v) were purchased from Liza Company, Egypt. Bleach cream was purchased from Ox Light, My Way Skin Clinic Limited, Egypt.

The standard solution of H_2_O_2_ was prepared by diluting 5.5 mL H_2_O_2_ to 500 mL with DDW. Phosphate buffer solution (PBS) was prepared *via* mixing 80 mL of Na_2_HPO_4_ 0.5 M (35.5 g Na_2_HPO_4_/500 mL DDW) and 30 mL of NaH_2_PO_4_ 0.5 M (30 g NaH_2_PO_4_/500 mL DDW) that has been reconstituted to 500 mL by DDW and adjusted to pH 7 by adding appropriate amounts of the 0.5 M NaH_2_PO_4_ solution.^35^

(0.04 M H_3_BO_3_, 2.04 g/100 mL) was mixed with (0.04 M H_3_PO_4_, 2.8 mL of 85% H_3_PO_4_/100 mL), and (0.04 M CH_3_COOH, 2.3 mL/100 mL) and set to the proper pH with NaOH to obtain Britton–Robinson (B. R) buffer.^36^

### 2.2. Instrumentation

An Adwa AD11P pH-meter (Romania) was used to measure pH values. The UV-Vis and luminescence measurements were carried out by Shimadzu UV-Vis (1601/PC, Japan) and a SCINCO FS/2 FluoroMate (Korea) spectrometers, respectively. Fourier-transform infrared (FT-IR) spectra were carried out by Nicolet™ iS™10 FTIR, Slovenia in the range of 400–4000 cm^−1^. The surface morphology images of FLS@MnO_2_ NS was done by scanning electron microscope (SEM), Hitachi and Transmission Electron Microscope (TEM, JEM-100CX II, USA). The phase crystallinity profile of FLS@MnO_2_ NS was studied utilizing a Philips X-ray diffractometer (1710 PW, Cu Kα radiation *λ* = 1.5405 Å, 40 kV voltage, 30 mA current, and 0.06° min^−1^ scanning rate, UK). Elemental analysis was performed using OXFORD INA energy dispersive X-ray instrument (EDX). The powder X-ray diffraction (PXRD) was scanned by Philips X-ray diffractometer PW 1710 supplied with 40 kV operating applied voltage, 30 mA current, 0.06° min^−1^ scanning rate in the 2*θ* range of (4–60°) and Cu Kα radiation (*λ* = 1.5405 Å).

### 2.3. Preparation of fluorescein modified manganese dioxide nanosheets (FLS@MnO_2_ NS)

**Scheme 1:**
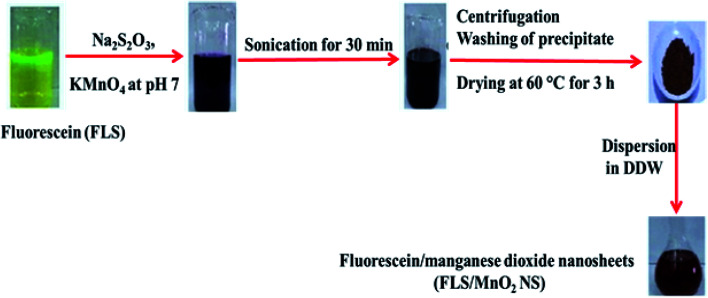
General steps for FLS@MnO_2_ NS synthesis.

The FLS@MnO_2_ NS were synthesized by a facile ultrasonic co-precipitation route. Scheme 1 shows a schematic illustration of the synthesis process. Briefly, FLS (0.01 g) and of Na_2_S_2_O_3_ (0.03 g) were dissolved in 200 mL PBS pH 7 and then 0.2 g KMnO_4_ was added to the solution at room temperature. The output mixture was sonicated for 30 min until the entire discharge of the pink color of permanganate and a brown colloid was formed. Subsequently, the brown colloid allowed to centrifugation (4000 rpm for 30 min) and separation of supernatant, and then the brown precipitate was collected and washed with DDW and absolute ethanol three times. After that, the precipitate was dried at 60 °C for 3 h in an electric oven and a purified 10 mg was dispersed in 10 mL DDW (1 mg mL^−1^) for further characterization and application.

### 2.4. Detection assay of H_2_O_2_ using FLS@MnO_2_ NS

350 μL FLS@MnO_2_ NS aqueous solution (0.35 mg L^−1^) and 500 μL various concentrations of H_2_O_2_ were added to 150 μL B. R. (pH 6.0). After 12 min at room temperature, the fluorescence spectra were observed under excitation of 495 nm.

### 2.5. Application to real samples

1.0 mL of oxygen water 10% v, v that is equivalent to 0.89 M, was diluted with DDW before application of the proposed fluorometric method.

0.1 g of bleach cream was dissolved in 20 mL of ethyl alcohol and stirred until complete dissolution, and the volume was completed to 100 mL calibrated flask. Different aliquots of the prepared solution were taken and analyzed by the proposed method.

Ibrahimia conduit water (Assuit, Egypt) was filtered four times through a qualitative filter paper to remove the insoluble matters, preserved in high-quality clean plastic container, and stored at 4 °C.^37,38^ The conduit water samples were spiked with known concentrations of H_2_O_2_ (5, 10, 15, 20, 25 μM) and were analyzed using the general procedure.

## 3. Results and discussions

### 3.1. Strategy of H_2_O_2_ detection using FLS@MnO_2_ NS

**Scheme 2:**
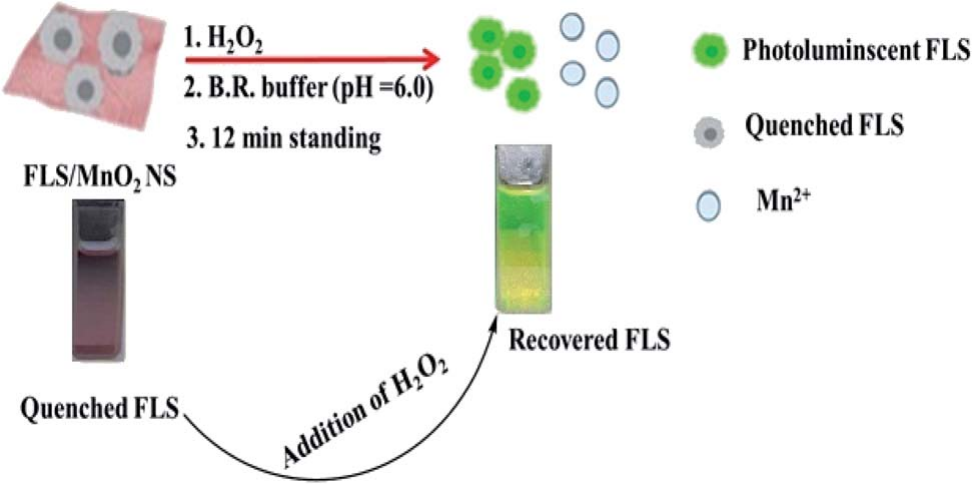
Principle for H_2_O_2_ detection using FLS@MnO_2_ NS.

The master plan for H_2_O_2_ determination was established on the capability to modulate the quenching of FLS luminescence that induced by MnO_2_ NS (Scheme 2).

An organic fluorophore, FLS as an energy donor, was adsorbed on the exterior surfaces of MnO_2_ NS, the nanosheets structure was primarily formulated thanks to the sonically reduction of permanganate by Na_2_S_2_O_3_ in PBS (pH 7). Since MnO_2_ NS has a wide absorption band ranging from 390 to 600 nm that remarkably interferes with the emission of FLS, the fluorescence of FLS can be efficiently faded by MnO_2_ NS.

**Fig. 1:**
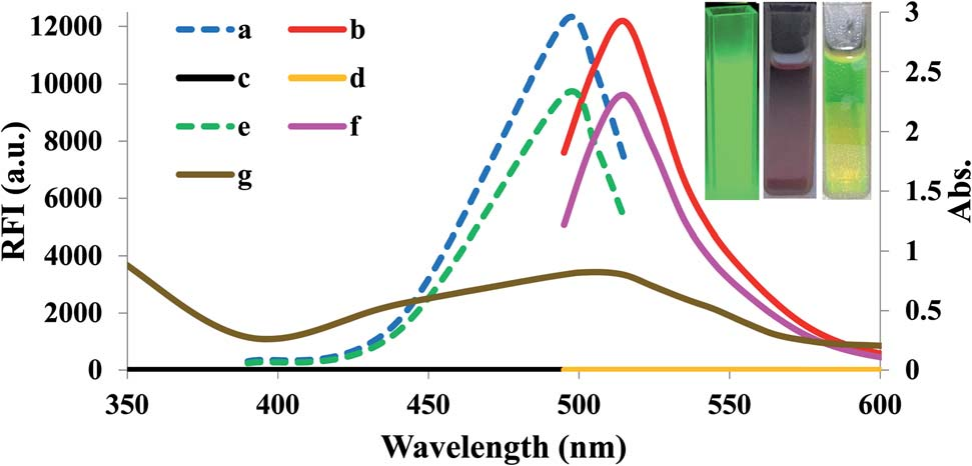
Overlay spectra of (a) excitation of unreacted FLS, (b) emission of unreacted FLS, (c) excitation of FLS@MnO_2_ NS (blank), (d) emission of FLS@MnO_2_ NS (blank), (e) excitation of FLS@MnO_2_ NS@20 μM H_2_O_2_, (f) emission of FLS@MnO_2_ NS@20 μM H_2_O_2_ while (g) is the absorbance of FLS@MnO_2_ NS. Inset: observable fluorescence images of unmodified fluorescein (left), FLS@MnO_2_ NS (middle) and FLS@MnO_2_ NS@20 μM H_2_O_2_ (right) under the portable UV lamp.

Small quantities of H_2_O_2_ can mediate the redox pathway by which MnO_2_ turned into Mn^2+^ leading to the decomposition of the MnO_2_ NS accompanied by fluorescence restoration (Fig. 1).

The aforementioned *in situ* redox can be exemplified as the following equation:^31,39^MnO_2_ + H_2_O_2_ + 2H^+^ → Mn^2+^ + 2H_2_O + O_2_

### 3.2. Characterization of FLS@MnO_2_ NS

**Fig. 2:**
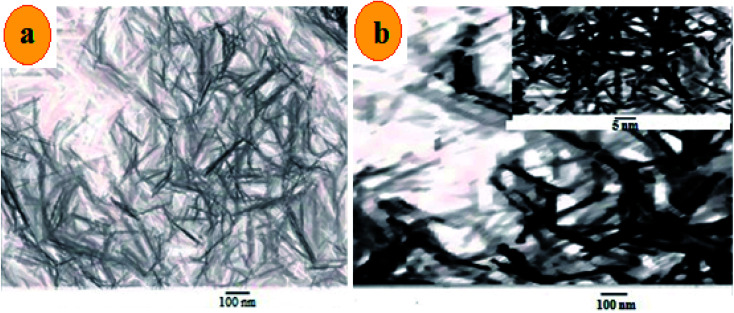
TEM images of (a) ultrathin MnO_2_ NS and (b) FLS@MnO_2_ nanocomposite at 100 nm and inset of (b) is a higher magnification.

The morphology of MnO_2_ NS and the formed FLS@MnO_2_ NS was characterized by TEM which revealed a lamellar nanostructure with large irregular folds, showing 2D morphology with a huge surface area. Furthermore, FLS/MnO_2_ NS were observed with thicker and less transparent flakes that may due to FLS conjugation MnO_2_ NS. These multiple folds provide high relevance due to their high surface area available for short transport paths for electrons and ions^40^ (Fig. 2).

**Fig. 3:**
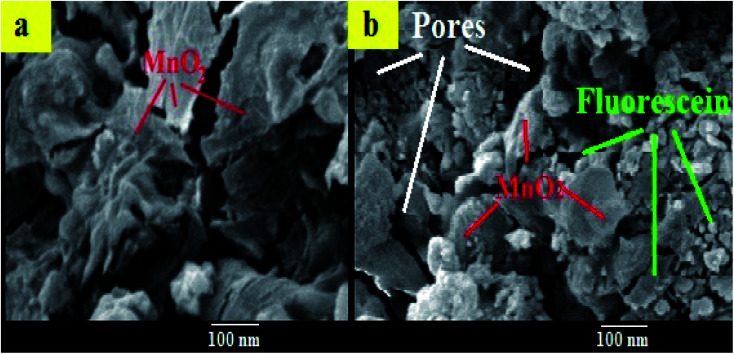
SEM images of (a) MnO_2_ NS (b) FLS@MnO_2_ NS.

Moreover, the surface morphology of the formed FLS@MnO_2_ NS was examined by SEM and the micrographs demonstrated high morphological purity. The film surface is compact and well wrapped with fine and disparate shaped grains (Fig. 3). Also, it is seen the surface looks highly porous which offers a large surface area. The high porosity and large surface area of films provide facile oncoming in the redox process and result in a high packing density of the active material. Nano-sized material limits the electron diffusion path which offers helpful support in the redox reaction. Such type of morphology leads to the porous volume, which provides the structural foundation for the high specific performance and the nanocomposite can be used as a cheap high potential catalyst in organic oxidation reactions.^41^

The phase purity and crystal structure of MnO_2_ NS were inspected by PXRD. As presented in Fig. 3, the diffraction peaks which appeared at 2*θ* = 12.7°, 18.1°, 28.8°, 37.5°, 42.1°, 57.2°, and 60.3° matched well with the diffraction peaks of (110), (200), (310), (211), (301), (600), and (521) crystal planes of α-MnO_2_, the PXRD pattern with sharp and intense peaks refers to a good crystallinity for the α-MnO_2_ in the composite.^42,43^

**Fig. 4:**
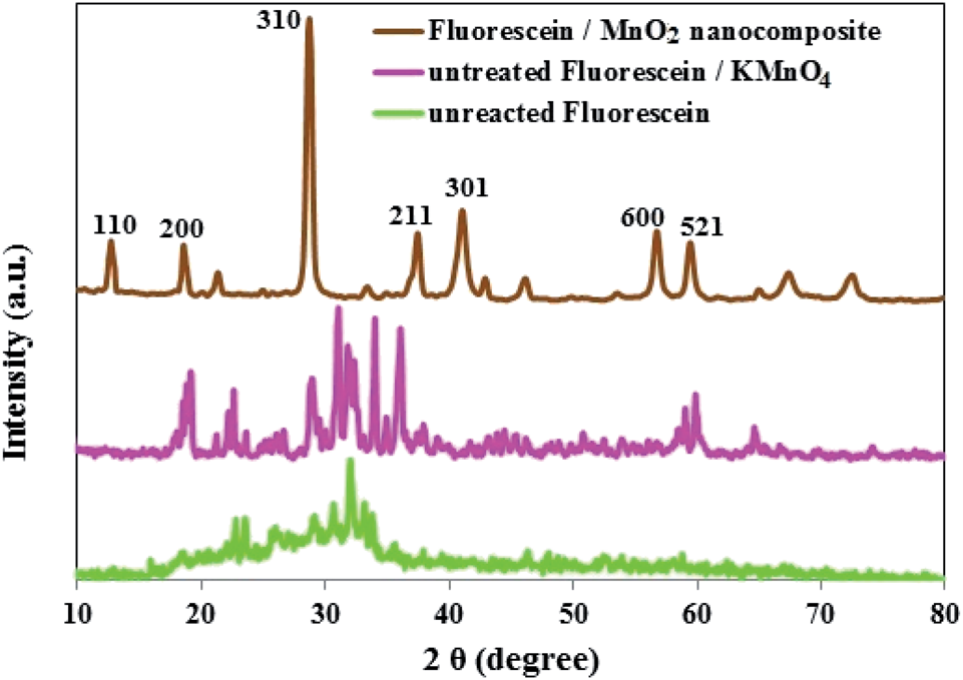
PXRD patterns of unreacted FLS, untreated FLS/KMnO_4_ and FLS@MnO_2_ NS.

Comparison of the PXRD patterns of unreacted FLS, untreated FLS/KMnO_4_ and FLS@MnO_2_ NS showed the absence of FLS peak in the FLS@MnO_2_ NS (Fig. 4), indicating an interaction between FLS and the MnO_2_ NS.^44^

The crosslinking of MnO_2_ NS and FLS was examined by FTIR spectra of MnO_2_ nanostructure (with and without FLS incorporation) and the results are shown in Fig. 1S.† The two characteristic bands between 600 and 400 cm^−1^ attribute to the stretching collision of O–Mn–O and were blue-shifted in the nanocomposite sample by FLS.^45^

Furthermore, the absorption peaks at 899 and 1009 cm^−1^ represent the surface –OH groups of Mn–OH for MnO_2_ NS which become wider in the nanocomposite product indicating the presence of FLS.^46^ In the high-frequency region, a broadband around 3400 cm^−1^ is observed which can be assigned to the stretching vibrations of adsorbed molecular water in MnO_2_ NS product and maximize thanks to stretching vibrations of the –OH group of FLS in nanostructure.^47^ The dominant peak at 1731 cm^−1^ can be assigned to the carbonyl stretching mode and the peaks at 1203 and 1117 cm^−1^ may be caused by –C–O–H stretching, implying the existence of residual hydroxyl groups^44^ (Fig. 1S†).

The EDX analysis of synthesized FLS@MnO_2_ NS showed the presence of Mn and O in the sample (Fig. 2S†). The chemical composition analysis using EDX confirmed the presence of Mn and O in the nanocomposite samples (Table 1S†) and was similar to the earlier studies of other researchers.^48^

UV-Vis spectra (Fig. 3S†) illustrate that unreacted FLS has a maximum absorbance of 490 nm. The pink-colored solution after adding KMnO_4_, before sonication, exhibits maximum absorbance at 540 nm. After sonication, the pink-colored product gradually converted into brown colloid indicating the synthesis of FLS@MnO_2_ NS that acquire blue shifting from 590 to 495 nm that interfere with FLS emission (515 nm) leading to the respected quenching effect.

### 3.3. Optimization of the experimental conditions

Many buffers were checked to show the performance of FLS@MnO_2_ NS and the best results were obtained using B. R. buffer. Fig. 4Sa† shows the effect of the media pH on the fluorescence enhancement of FLS@MnO_2_ NS in the presence of H_2_O_2_.

A rise in pH from 3 to 5 results in the increased fluorescence enhancement efficiency of the FLS@MnO_2_ NS at 515 nm after the addition of H_2_O_2_, further increase in pH from 5 to 8 leads to a plateau, whereas a further increase in pH from 8 to 10 leads to a gradual decrease. Consequently, we selected 6.0 as the optimal pH for our study using B. R. as a buffering system.

The effect of the concentration of FLS@MnO_2_ NS on the fluorescence enhancement efficiency is displayed in Fig. 4Sb.† The fluorescence enhancement efficiency progressively increased with the concentration up to 0.3 mg mL^−1^. Exceeding that, the fluorescence intensity didn't affect. Therefore, 0.35 mg mL^−1^ was used as the optimal concentration for further performance.

The influence of incubation time on the fluorescence intensity of the system is shown in Fig. 4Sc.† The fluorescence enhancement became slow until reaching a steady state at 12 min. A further increase of time didn't lead to any further perceptible enhancement. So, 12 min was chosen as the optimum incubation time.

### 3.4. Calibration plot, LOD and LOQ

**Fig. 5:**
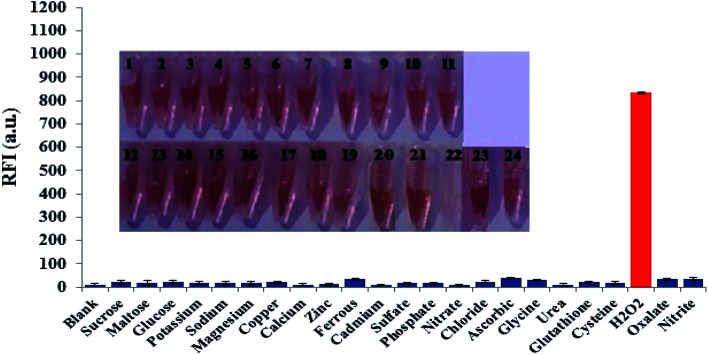
Fluorescence responses of FLS@MnO_2_ NS in the presence of different interfering species (300 μM of each) at the selected conditions. Inset: photographs (from 1 to 24) corresponding to *X*-axis species of the diagram (from left to right). The concentrations of H_2_O_2_ (2 μM), error bars represent the standard deviations of three repetitive experiments.

After successfully fabricated the FLS@MnO_2_ NS, the chance of H_2_O_2_ detection has then explored; we applied the developed procedure and inspected the fluorescence response signals at serial diverse H_2_O_2_ concentrations. At first, the fluorescence intensity of blank (replacing H_2_O_2_ loaded sample with double-distilled water) is nearly negligible at the selected conditions while it was progressively increased with H_2_O_2_ incorporation. As the concentration of H_2_O_2_ increases, the fluorescence intensity increases (Fig. 5Sa†).

The addition of 30 μM H_2_O_2_ led to a considerable enhancement, which indicated an almost complete recovery of free unreacted FLS. The recovery of fluorescence was related to the reduction of MnO_2_ which led to the degradation of the MnO_2_ NS induced by H_2_O_2_ associated with the liberation of FLS. The fluorescence intensity values at 515 nm were rectilinear with the H_2_O_2_ concentrations in the range of 40 nM to 30 μM, (*R*^2^ = 0.998) (Fig. 5Sb†) with a mean relative standard deviation (RSD) of 2.6%. Besides, the LOD [in terms of 3.3× standard deviation of the regression line (*σ*)/slope(*S*)] was 7 nM and the LOQ [in terms of 10 × (*σ*)/(*S*)] was 21 nM.

### 3.5. Selectivity study

Possible interfering matters including various chemical, environmental and biological species were incubated with a solution of FLS@MnO_2_ NS at the selected experimental conditions. As shown in Fig. 5, only H_2_O_2_ can dissociate the nanocomposite, accompanied by discoloration of the brown, accordingly regenerate and enhance the fluorescence intensity. This investigation revealed that 300 μM of a wide range of electrolytes and weakly reducing bio-agents didn't produce notable optical responses as well as didn't degrade the nanostructure as the color almost didn't change. It is worth mentioning that although some reports deduced the reduction of MnO_2_ NS by ascorbic acid but in our study the proposed method not affected significantly by ascorbic acid that may be attributed to the low acidity that not sufficient to oxidize ascorbic acid by MnO_2_ NS.

### 3.6. Real samples analysis

**Fig. 6:**
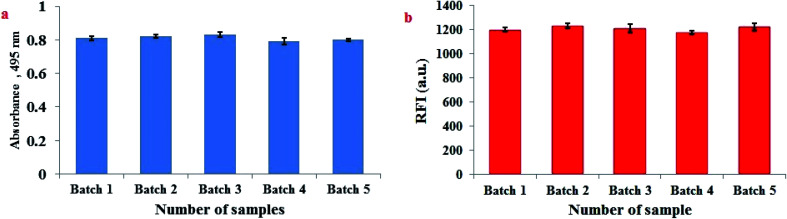
Homogeneity of FLS@MnO_2_ NS and reproducibility of the synthesis procedure assessed by (a) spectrophotometric determination at 495 nm of five independent batches and (b) fluorescence detection of 3 μM H_2_O_2_ at five independent batches of FLS@MnO_2_ NS.

To test the analytical validity of this approach, the method was applied for the determination of H_2_O_2_ in pharmaceutical oxygen water, cosmetic cream and natural water using the procedures described in Section 2.5. As can be seen in Fig. 6S,† the emission spectrum for the analyzed pharmaceutical, cosmetic and natural samples is very identical to that exhibited in Fig. 5Sa.† This displays that the species existing in the inspected samples do not interfere in the estimation of H_2_O_2_ by the suggested approach. The analysis details of the real sample are signalized in (Table 1)

**Table 1:** Determination of H_2_O_2_ in real samples using the proposed method and other reported method

Samples	Taken (μM)	Found^a^ (μM)	Reported method^49^ (μM)	*t*-Value	Recovery (%)	RSD (%)
H_2_O_2_, oxygen water	5.0	4.3 ± 0.019	4.5	0.12	86.8 ± 4.2	3.2
	10.0	10.1 ± 0.023	9.9	0.17	101 ± 2.3	1.4
	15.0	14.1 ± 0.015	14.2	1.22	95.6 ± 3.5	4.2
	20.0	21.3 ± 0.022	20.1	1.3	106.5 ± 4.3	2.7
	25.0	24.3 ± 0.034	24.7	0.25	97.2 ± 2.4	3.9
OX light, bleach cream	5.0	5.1 ± 0.024	5.8	0.12	101.7 ± 2.1	4.3
	10.0	10.6 ± 0.012	10.9	0.38	105.8 ± 1.1	3.8
	15.0	15.5 ± 0.012	15.9	1.13	103.3 ± 3.3	4.6
	20.0	20.2 ± 0.042	20.4	1.23	101.1 ± 2.9	3.5
	25.0	24.3 ± 0.031	25.3	2.14	97.4 ± 3.6	2.9
Ibrahimia conduit water	5.0	5.3 ± 0.026	5.2	1.24	98.1 ± 2.5	1.3
	10.0	10.3 ± 0.034	10.4	0.45	103 ± 0.9	3.7
	15.0	15.5 ± 0.021	15.8	1.42	103.3 ± 2.1	2.9
	20.0	21.2 ± 0.008	20.8	0.89	106 ± 4.2	2.4
	25.0	25.8 ± 0.003	24.8	2.11	103.2 ± 3.2	1.8

a Mean of three determinations.

The recoveries of H_2_O_2_ fall in 86.8–106.5%, implying that the synthesized nanoprobe can be efficiently used to determine H_2_O_2_ in real samples, the low value of RSD% denotes the precision and feasibility of this method for determination of the respected analyte in real samples. To approve the accuracy of the proposed method, we statistically compare the results deduced by the current approach and other reported fluorometric method for the determination of H_2_O_2_ that was intended to be used to oxidize non-fluorescent coumarin to highly fluorescent 7-hydroxycoumarin.^49^ As can be seen from Table 1, all the calculated t-values are below the critical *t* value of 2.571 for 95% confidence level and 5 degrees of freedom. Therefore, the accuracy of the method for the determination of the studied analytes is confirmed.

### 3.7. Stability of FLS@MnO_2_ NS

The stability potential of the nanoprobe is of important value from an analytical point of view. The more stable a probe is, the more is its capacity for broad applications. Subsequently, the time-stability of the FLS@MnO_2_ NS was examined after storing under normal conditions. After storing for one month, the material was collected by centrifugation and washed with double distilled water and ethanol, then dried in an oven at 60 °C for 3 h and measure the absorbance signal of the dispersed solution. Fig. 7S† indicated that the absorbance response of FLS@MnO_2_ NS was decreased slightly and no obvious change in color or morphology after storing for one month under normal conditions.

### 3.8. Determination of glucose *via* enzymatic degradation to H_2_O_2_

The proposed approach also proceeded for glucose sensing to examine the generality of the FLS@MnO_2_ NS. From the standpoint of biochemistry, glucose can be catalytically oxidized by glucose oxidase enzyme and disintegrated into gluconic acid and H_2_O_2_, thus, glucose can be detected *via* the sensing of bio-enzymatically developed H_2_O_2_. According to reported method,^50^ and with slight modification, different concentrations of glucose solution (5, 10, 15, 20 and 25 μM) was successively added to 100 μL B. R. buffer (pH = 6.0) and 100 μL of glucose oxidase enzyme, followed by successively mixing and incubation at room temperature for 5 min to obtain H_2_O_2_. Then, the dispersion of the prepared FLS@MnO_2_ NS (350 μL) was added into the above solution. The resulting mixture was successively incubated at room temperature for 12 min for the further fluorescence determination. As shown in Fig. 8S,† as the glucose concentration increased, the fluorescence intensity increased. The fluorescence intensity at 515 nm was rectilinear correlated to the glucose concentration (5–25 μM, *R*^2^ = 0.9784) proving that the FLS@MnO_2_ NS is generalizable and can be utilized to detect various H_2_O_2_ generating syntheses.

### 3.9. Comparison of the proposed method with other methods

Comparing the results in the proposed method with other published methods; from Table 2, it can be seen that the FLS@MnO_2_ NS can serve as a probe for the detection of H_2_O_2_ in a more wide concentration range, and can determinate them in a low concentration.

**Table 2:** Several reported sensors for the determination of H_2_O_2_

Method	Range (M)	LOD (M)	RSD%	Ref.
Colorimetry	2 × 10^−5^–8 × 10^−5^	1 × 10^−5^	2.4	6
Colorimetry	1 × 10^−5^–8 × 10^−5^	2 × 10^−6^	4.1	7
HPLC	2 × 10^−7^–1 × 10^−4^	1 × 10^−7^	2.7	13
GC	1 × 10^−6^–1 × 10^−4^	6 × 10^−7^	4.7	15
Electrochemical	4 × 10^−5^–4 × 10^−3^	1 × 10^−5^	3.4	51
Electrochemical	1 × 10^−6^–1 × 10^−3^	1 × 10^−7^	4.3	52
Chemiluminescence	5.8 × 10^−7^–4.7 × 10^−5^	2.6 × 10^−7^	4.5	53
Fluorimetry	2 × 10^−8^–2 × 10^−5^	5 × 10^−9^	2.3	54
Fluorimetry	1 × 10^−6^–3.5 × 10^−4^	9 × 10^−7^	2.9	31
Fluorimetry	1 × 10^−6^–2 × 10^−4^	3.3 × 10^−7^	3.0	32
Fluorimetry	4 × 10^−6^–1 × 10^−4^	0.87 × 10^−6^	2.9	55
Fluorimetry	4 × 10^−8^–3 × 10^−5^	7.5 × 10^−9^	2.6	This work

It is worth to mention that the proposed fluorometric method, if compared to other reported methods, has low detection limit and low % RSD, indicating higher sensitivity and reliability of the proposed fluorometric method for analysis of H_2_O_2_. Moreover, the applicability of the fluorometric method was extended for the determination of the target analyte in different matrices.

### 3.10. Homogeneity of FLS@MnO_2_ NS and reproducibility of the synthesis procedure

The homogeneity of FLS@MnO_2_ NS and reproducibility of synthesis procedure were performed through analysis of five independent batches of FLS@MnO_2_ NS spectrophotometrically at 495 nm. Moreover, 3 μM H_2_O_2_ was repeatedly assayed by the proposed fluorimetric method in five separate sets using five independent batches of FLS@MnO_2_ NS. The % RSD did not exceed 3.89% which confirms that the synthesis procedure of FLS@MnO_2_ NS is homogenous and reproducible (Fig. 6).

## 4. Conclusion

In summary, we have developed FLS@MnO_2_ NS platform *via* an uncomplicated one-step solution-phase passageway, by the reduction of potassium permanganate with sodium thiosulphate at room temperature with no aid of catalysts or templates and demanding no expensive and precise equipment, guarantees higher purity of the products, exceedingly diminishes the production cost and hence offers a great chance for analytical scale-up preparation of nanostructured materials. As a further matter, it is attractive that the as-synthesized FLS@MnO_2_ NS can be used as an effective nanoprobe for the detection of H_2_O_2_ with higher selectivity and sensitivity and applied to determine the respected analyte in real samples with acceptable results. The proposed nanoprobe has great potential for analytical and clinical investigation. In the meantime, this nanostructure is also generalizable and can be readily continued to sense several H_2_O_2_ generating substances as a logic gate application. The proposed protocol may provide a new insight to develop low-cost and sensitive methods for food, environmental, biological and clinical diagnostics applications.

## Conflicts of interest

The authors declare that they have no known competing financial interests or personal relationships that could have appeared to influence the work reported in this paper.

## Supplementary Material

RA-010-D0RA01980A-s001
