# Supplementary material for: One pot fabrication of fluorescein functionalized manganese dioxide for fluorescence “Turn OFF–ON” sensing of hydrogen peroxide in water and cosmetic samples
Source: RSC Adv. 2020 May 5;10(30):17506–14. doi: 10.1039/d0ra01980a (PMC9053515; doi:10.1039/d0ra01980a)
Supplement: RA-010-D0RA01980A-s001 [file RA-010-D0RA01980A-s001.pdf]

Electronic Supplementary Material (ESI) for RSC Advances.
This journal is © The Royal Society of Chemistry 2020

Supporting information

One pot fabrication of fluorescein functionalized manganese dioxide for fluorescence "Turn OFF-ON" sensing of hydrogen peroxide in water and cosmetic samples

Hassan Refat H. Ali[a], Ahmed I. Hassan[b], Yasser F. Hassan[b], Mohamed M. El-Wekil[a*]

[a] Department of Pharmaceutical Analytical Chemistry, Faculty of Pharmacy, Assiut University, Assiut 71526, Egypt.

[b] Department of Pharmaceutical Analytical Chemistry, Faculty of Pharmacy, Al Azhar University, Assiut 71526, Egypt.

***Corresponding author:** mohamed.mohamoud@ymail.com

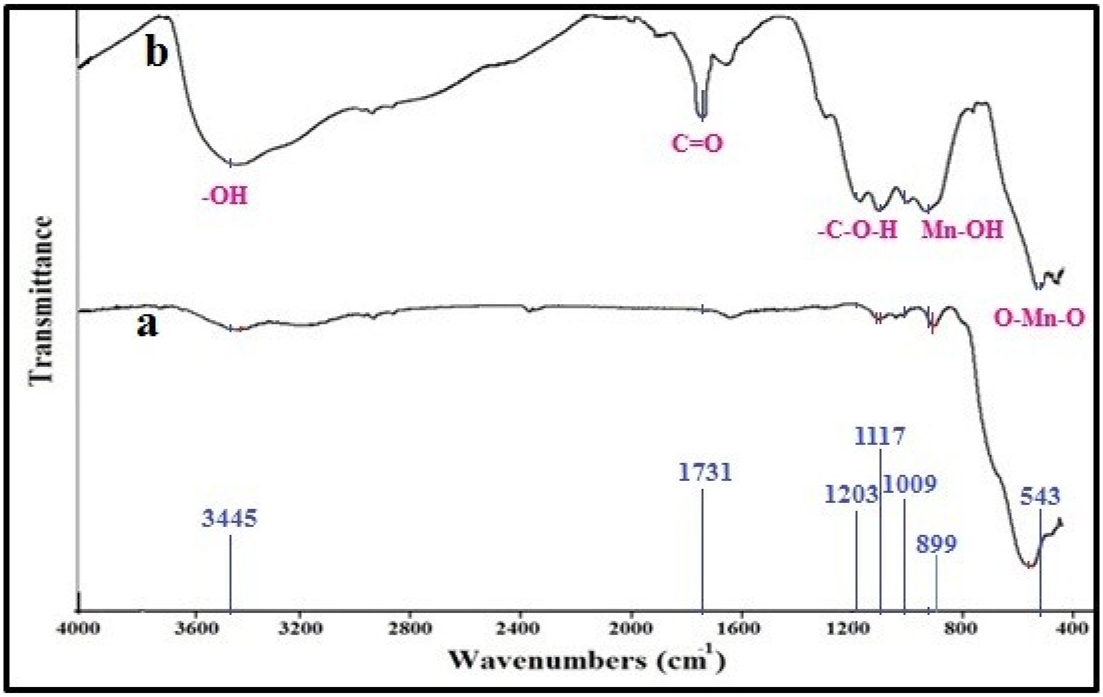

Figure 1S: FT-IR spectra of (a) MnO_2 NS (b) FLS@MnO_2 NS

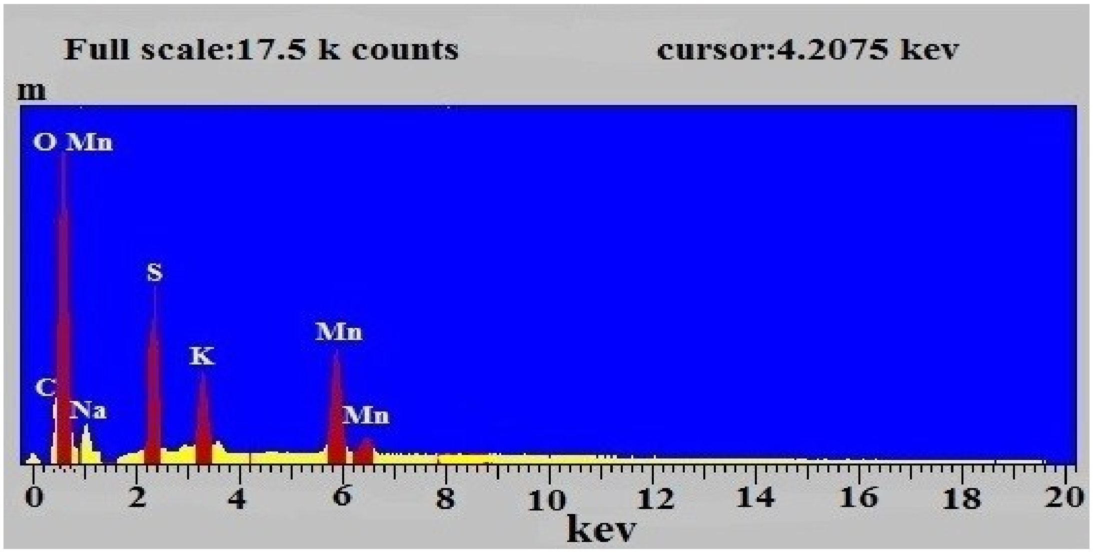

Figure 2S: EDX analysis of the synthesized FLS@MnO_2 NS.

Table 1S: EDX analysis of elemental composition of synthesized FLS@MnO_2 NS.

Element	C K	O K	Na K	S K	K K	Mn K	Total
Weight %	3.12	31.23	1.97	9.41	7.22	47.05	100.00

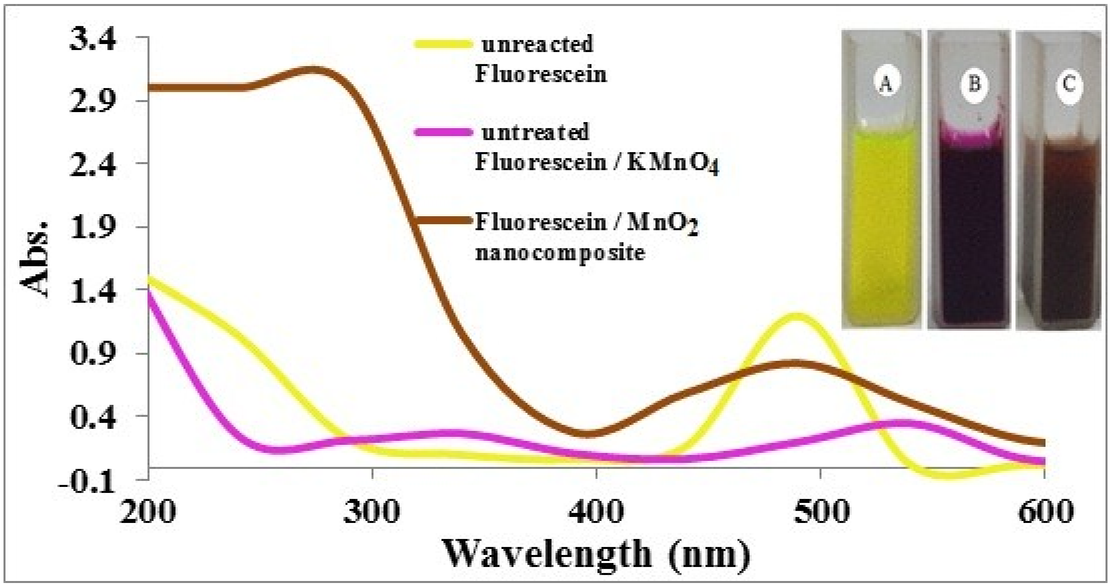

Figure 3S: Overlay UV-VIS spectra of: unreacted FLS, untreated FLS / $KMnO_4$ and FLS@MnO_2 NS. Inset: observable color images of (A) unreacted FLS (B) untreated FLS/$KMnO_4$ (C) FLS@MnO_2 NS

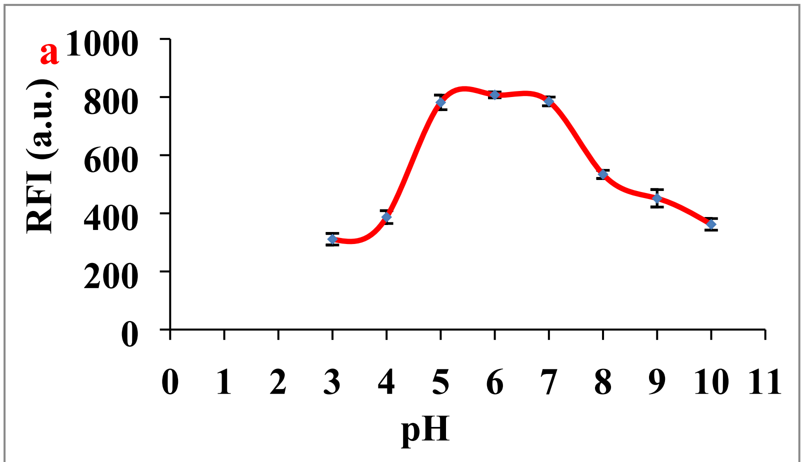

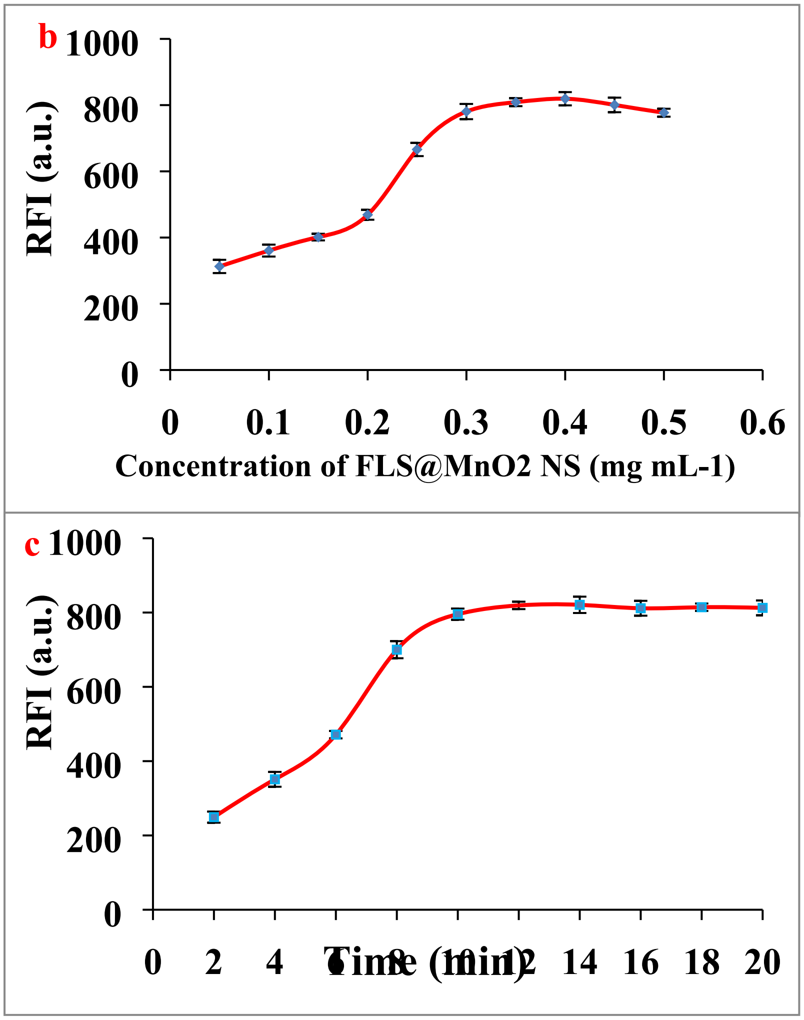

Figure 4S.The effect of (a) pH of B.R. buffer, (b) concentration of FLS@MnO_2 NS (c) incubation time on fluorescence enhancement efficiency of the experimental procedure containing 2 μM H_2O_2

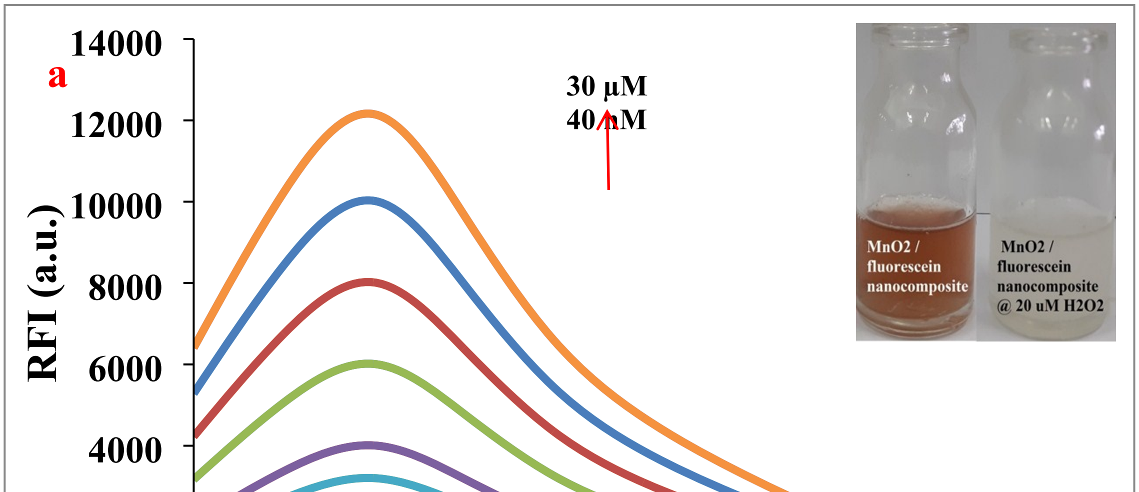

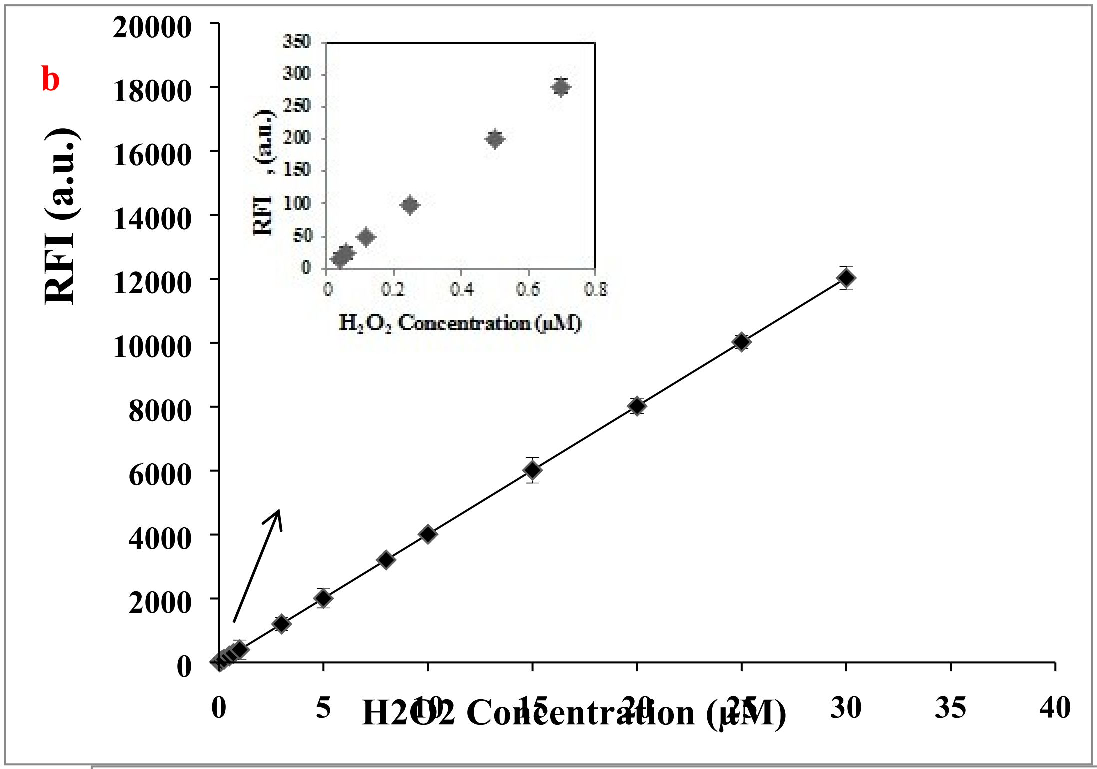

Figure 5S[illegible] H_2O_2 of different concentrat[illegible] 515 nm against the H_2O_2 conc[illegible]nts.

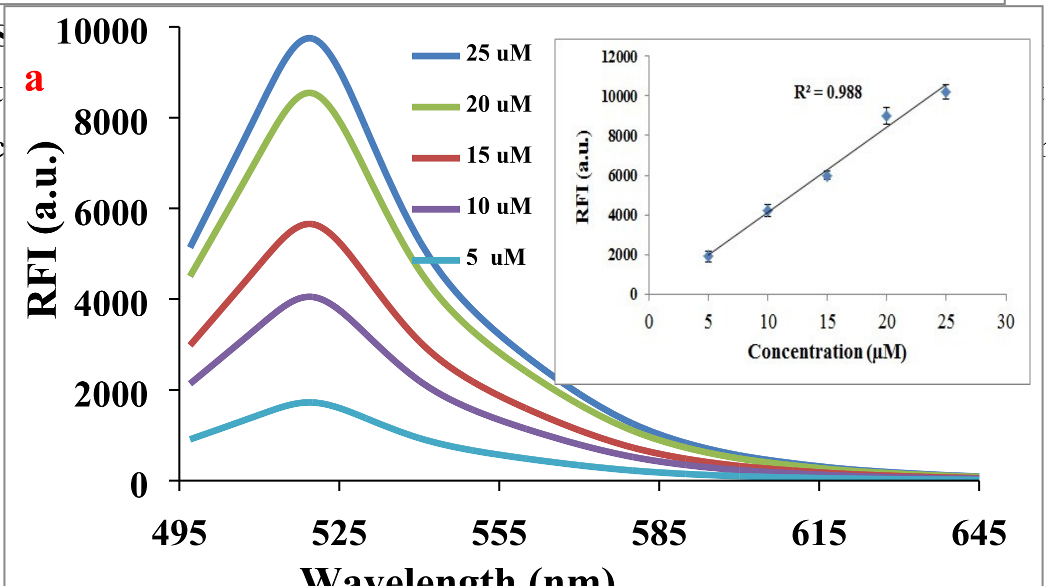

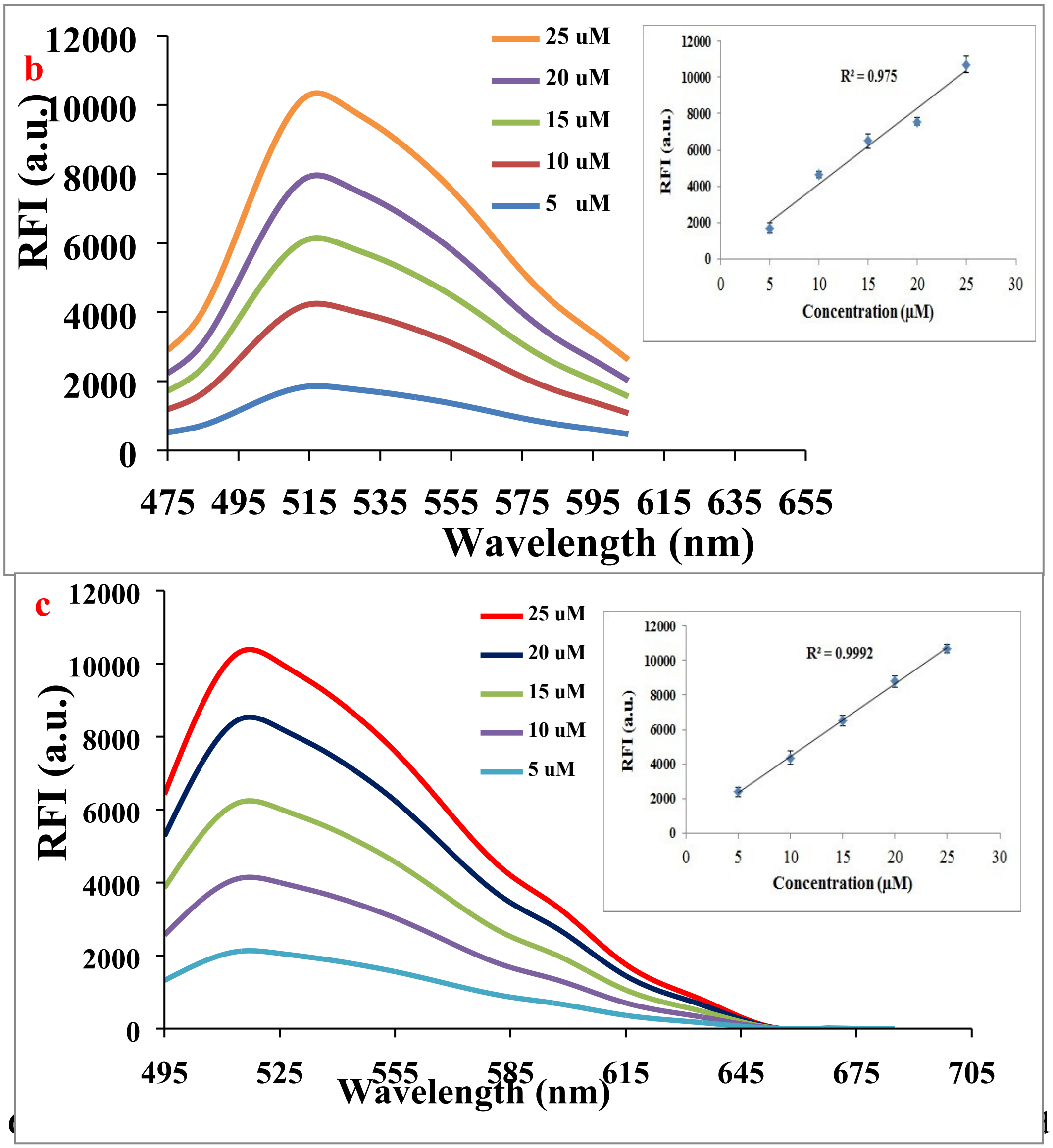

Figure ([illegible] l oxygen water sample (a), cosmetic cream sample (b) and natural water sample (C) using the proposed nanosystem at the optimum conditions.

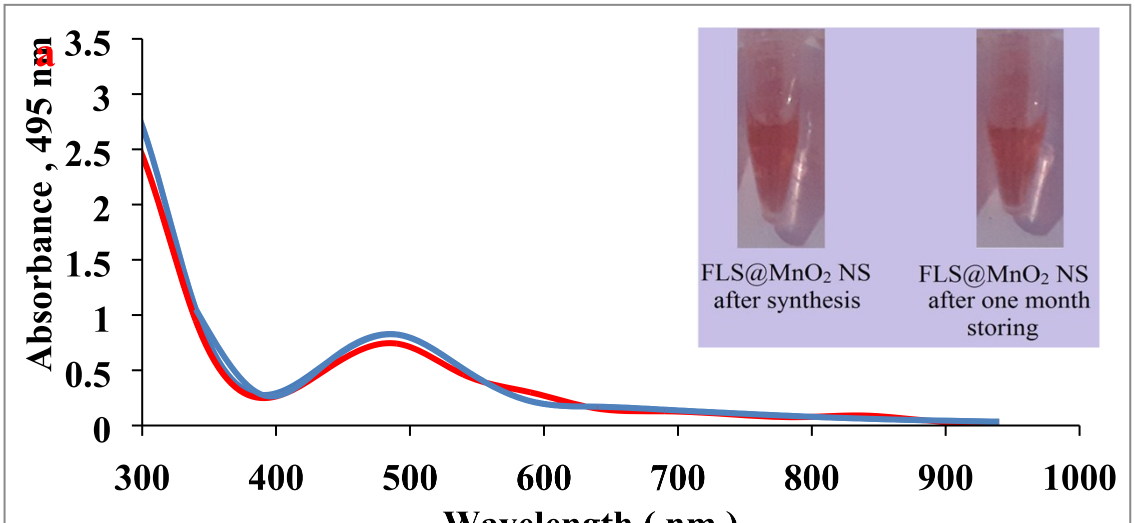
a
Absorbance , 495 nm
3.5
3
2.5
2
1.5
1
0.5
0
300
400
500
600
700
800
900
1000
FLS@MnO2 NS after synthesis
FLS@MnO2 NS after one month storing

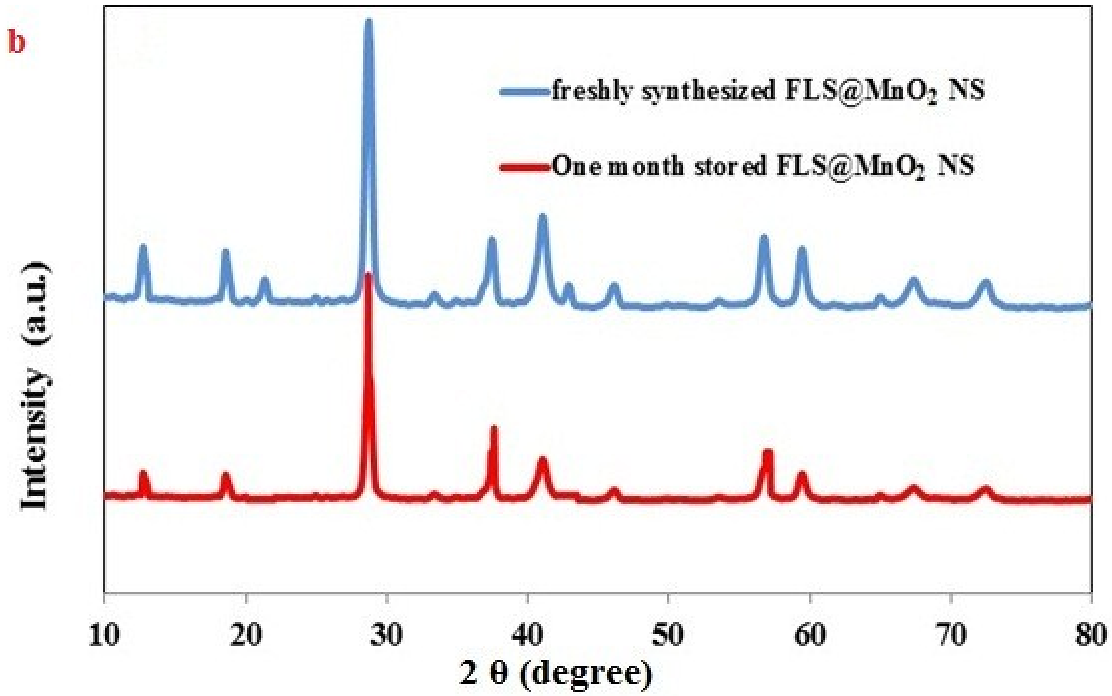

Figure 7S: (a) absorption spectra of the freshly synthesized FLS@MnO_2 NS (blue) and the one month stored FLS@MnO_2 NS (red), inset: photograph images of color change (b) PXRD charts.

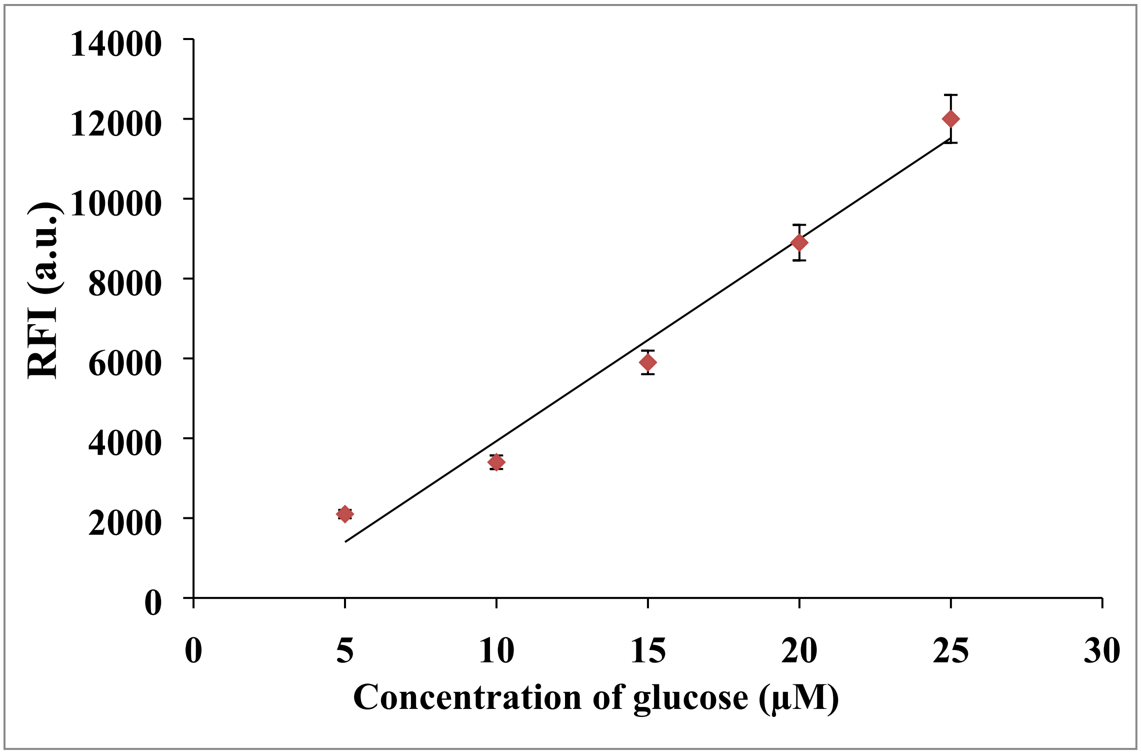

Figure 8S: Plot of the fluorescence intensity at 515 nm against the glucose concentration using the proposed nanoprobe with the intended modification.
